# Role Perception of Occupational Therapists in Education Systems: Self-Efficacy and Employability Skills

**DOI:** 10.1155/2021/5531224

**Published:** 2021-11-24

**Authors:** Yael Fogel, Liron Lamash

**Affiliations:** ^1^Department of Occupational Therapy, Ariel University, Ariel 4077625, Israel; ^2^Department of Occupational Therapy, University of Haifa, Haifa 3498838, Israel

## Abstract

Although the main framework for occupational therapy includes delivery of services in educational institutions with students with disabilities, little is known about how the occupational therapists perceive their role in this specific system. This research examines an integrated conceptual model wherein self-efficacy explains the occupational therapists' role perception with employability skills as a mediator. Participants, 147 occupational therapists working in educational systems, completed an online demographic questionnaire, the Perception Questionnaire for Occupational Therapists in the Education System, General Self-Efficacy Questionnaire, and Employability Key Questionnaire. The proposed model was analyzed by the structural equation model (SEM) using AMOS software. The SEM provided excellent goodness of fit indices, *χ*^2^(24) = 40.49; *p* = .019; NFI = .93; CFI = .97; RMSEA = .07; SRMR = .05, and explained 40% of the variance in role perception. These findings highlight employability skills as the primary contributor that affects occupational therapists' role perception. Self-efficacy and employment skills influence how occupational therapists working in education systems perceive their roles; thus, employment skills should be included in professional training and development courses. This study has implications for occupational therapists working on the education system to understand the meaningful effects of employability skills as critical to developing and improving their role perception.

## 1. Introduction

The range of activities required of occupational therapists who work in education systems includes supporting the educational environment, academic achievement, and social participation of students with disabilities [1, 2]. Occupational therapists identify the students' strengths, as well as factors that may interfere with their learning and participation in the context of educational activities, routines, and environments [1]. For occupational therapists, working in the educational system raises some challenges. In Truong and Hodgetts's [3] study, teachers indicated three themes that demonstrate some of those challenges: teachers' confusion over the occupational therapists' role and scope of practice, teachers' desire for more reciprocal communication and more frequent opportunities to collaborate with occupational therapists, and teachers' desire for occupational therapists to have increased awareness of the needs and constraints of classroom contexts.

Recently, Bolton and Plattner [4] indicated continued discrepancies between occupational therapists' and teachers' understanding and use of occupational therapists' roles in the school setting. Their study highlighted concerns about how school-based professionals could make appropriate service referrals if they did not fully understand or define the role of occupational therapy.

Such organizational *role definition* would clarify the organization's employee requirements [5]. However, little research exists about the social-organizational and psychological dimensions related to associated viewpoints, attitudes, understandings, approaches, or expectations—that is, the *role perception* [5, 6]—of occupational therapists who work in education systems. A new study found that teamwork in the education system, relationship with parents, professional abilities, and a broad connection to the profession was the main factors of occupational therapists' role perception in the educational environment [7]. Thus, our study is aimed at examining how general employment-skill and self-efficacy variables affect the development of role perception among these occupational therapists.

Role perception is one of the most vital components in understanding an individual's concatenation in the workplace. Role perception and its actualization are a combined expression of the individual's psychological dimension and the social-organizational dimension in which they act [6]. It is subjective: two workers with identical role definitions can have entirely different role perceptions [5]. The definitions deal with internal and external factors that affect role perception. Internal factors are the individual's personality, attitude, values, beliefs, experiences, needs, and motives; external factors include the environmental stimuli's dimensions, frequency, intensity, and contrast [8].

Evidence from the literature on the general work world indicates that individuals' role perceptions and abilities to fulfill job responsibilities relate to their employability [9, 10]. *Employability* refers to gaining and maintaining employment or obtaining new employment if required [11]. *Employability skills* are the knowledge and skills needed to obtain and retain the job [12]. Smith and Krüger [13] listed such competency areas as personal qualities, relationships with others, maturity, health and safety habits, job commitment, problem-solving, decision-making, communication, and task-related skills.

Employment skills of occupational therapists in general, and specifically in education systems, have barely been studied. An up-to-date literature review revealed that employers of occupational therapists seek a range of qualities from staff, most frequently placing importance on experience, communication, interpersonal skills, and the ability to work within a team and in the field of practice [14].

Adam et al. [15] found that the most critical skill for successful practice as an occupational therapy clinician is communication—including interpersonal, interprofessional, and presentation. Although such communication would be essential in every field of practice, communication with a complex range of stakeholders and a job-specific language, as in occupational therapy, requires more workplace negotiation and mediation skills.

In accordance with self-efficacy theory (e.g., [16, 17]), we speculated that self-efficacy would be an important factor in the employment of occupational therapists. The concept of *self-efficacy* concerns mainly a person's belief in their competencies and capabilities to obtain employment [18]. According to the social-cognitive theory (the conceptual infrastructure of self-efficacy), individuals who do not believe they can attain the desired results with their behavior are less willing to convert the behavior into performance [18]. Bandura [16] asserted that self-beliefs are a major determinant of performance and that individuals vary in their beliefs about their level of control over actions needed to attain successful outcomes.

There is no clear answer in the literature regarding the relationship between those concepts. Do individuals with high self-efficacy perceive themselves as more employable, or does employability strengthen self-efficacy? According to Berntson et al. [19], employability predicted subsequent self-efficacy among a sample of individuals between the ages of 26 and 51 drawn from the Swedish population. However, Cmar et al. [20] found that self-efficacy was a significant predictor of employment, particularly for younger people and those who experienced recent significant vision loss.

**Figure 1:**
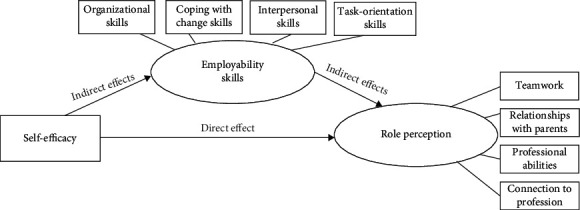
Self-efficacy, employability skills, and role perception conceptual model.

Our study assumes that high self-efficacy and employability skills may improve the role perception of occupational therapists working in education systems.  Figure 1 depicts the proposed theoretical model underlying the direct and indirect factors affecting that role perception. This conceptual model assumes that increased self-efficacy among occupational therapists will improve the employability skills that increase their role perception.

## 2. Material and Methods

### 2.1. Participants

We recruited study participants through online advertisements aimed at occupational therapists working in preschool to higher education systems. A sample size of 115 participants was determined using G^∗^Power software guidelines, considering the medium effect size of 0.25, power = 90, and *α* = 0.05 [21]. The sample included 147 participants aged 23 to 65 years (*M* = 38.37, SD = 10.13). Their professional seniority ranged from 0.5 to 35.0 years (*M* = 12.75, SD = 9.23), and their full-time equivalency (FTE) ranged from 17% to 121% (*M* = 75.23, SD = 25.94). Only 33.3% were members of professional organizations, and 42.9% had advanced degrees.

### 2.2. Instruments

#### 2.2.1. Demographic Questionnaire

This study's demographic questionnaire included questions related to age, gender, employment status, professional seniority, FTE, advanced degrees, and membership in professional organizations.

#### 2.2.2. Role Perception Questionnaire for Occupational Therapists (RP-OT) in the Education System

The RP-OT was developed to evaluate role perception among occupational therapists working in education systems worldwide. It has content and construct validation and was found to be reliable (all items *α* = .88; factors *α* = .64–.81) [7]. Respondents rate how much each statement reflects their role on a Likert scale from 1 (*slightly*) to 7 (*very much*). Based on exploratory factor analysis (EFA), we performed principal factor extraction and varimax rotation to examine the underlying construct validity. The 20 RP-OT items had high internal consistency reliability (*α* = .88) following EFA: factor 1 (teamwork in the education system, seven items) had .80, factor 2 (relationships with parents, four items) had .78, factor 3 (professional abilities, six items) had .81, and factor 4 (wide connection to the profession, three items) had moderate internal consistency reliability of .64.

#### 2.2.3. Employability Key Questionnaire (EKQ)

The EKQ includes a list of employability skills that human resource departments rated as desirable in job candidates [22]. Participants indicate the extent to which they consider their own abilities on a Likert scale from 1 (*very slightly*) to 7 (*very much*). The questionnaire construct validity was analyzed using Varimax factor analysis. The variance explained by the four factors was 57% (eigenvalue > 1). Accordingly, the EKQ includes 36 items in four skill clusters reflecting critical skill categories: (a) *organizational skills*, which is conducting oneself in the organization, includes oral, written, and basic computer communication skills; negotiation skills; assertiveness; initiative; and ability to access information necessary to accomplish organizational objectives; (b) *interpersonal skills*, which is communicating with colleagues and other personal attributes that contribute to positive work relationships, such as listening, giving sensitive feedback, aesthetic appearance, and effective teamwork; (c) *coping with change skills* that reflect adaptability to the unrelenting changes characterizing organizations today, such as openness to challenges and accommodating organizational change; and (d) *task-orientation skills*, referring to personal day-to-day, practical, results-oriented functioning (e.g., achieving objectives, managing time, planning work, working autonomously, being resourceful, and making do with available resources). An average score is calculated for each skill cluster. Scores of 5.6 to 7.0 (high) represent skill levels that help successful integration into the labor market; 3.6 to 5.5 (moderate) are usually high enough to find good jobs but developing some strengths could be beneficial in the long run; and 1.0 to 3.5 (low) represent levels with which it is possible to find a job but the position may not be as satisfying as desired. In the current study sample, a high internal consistency reliability was present for all EKQ items (*α* = .94) and categories (*α* = .81–.85).

Concurrent validity of the EKQ was found with criteria of occupational adjustment (such as an internal-control code versus an external-control focus at work, a sense of self-efficacy regarding job search, and positive self-perceptions). Moderate to high correlations were found (*r* = .24 − .50; *p* < .001). Predictive validity was found, such that the higher participants assessed their organizational and task-orientation skills, the longer was their duration of employment [22].

#### 2.2.4. General Self-Efficacy Questionnaire (GSE)

The GSE's purpose is to assess a person's sense of self-efficacy as they perceive it and as expressed in their life in general [23]. Respondents indicate how correctly each of 14 statements relates to them on a Likert scale from 1 (*not at all*) to 5 (*very much*). The total score is the average of the item scores; higher scores indicate higher sense of self-efficacy. The internal consistency has been found to be very high (.87–.95) [24], with content validity of .88 to .98 and predictive validity of the leadership trait of .94 [25]. In the current study sample, the internal consistency reliability using Cronbach's alpha formula was .92.

### 2.3. Procedure

The (blinded) Ethics Committee (AU-HEA-YF-20191030) and the Education Ministry Ethics Committee (No. 1000 990) approved this study. With the assistance of Occupational Therapy Supervisors in the National Ministry of Education, an email explaining the research and its requirements was sent to relevant occupational therapists. Interested participants signed printable online consent forms and then were provided links to online questionnaires.

We tested two proposed theoretical models. Only one, a structural equation model (SEM), fitted the data well and successfully tested both the direct and mediated variables. Self-efficacy is the observed (measurable) variable, and employability skills and pole perception are the theoretical latent variables.

### 2.4. Data Analysis

Using the bootstrapping method, we conducted the SEM to examine the conceptual mediation model. The bootstrapping procedure's value lies in its ability to process repeated simulations of subsamples from an original database. With this, we could assess the parameter estimate stability and report their values with greater accuracy. Bootstrapping estimates each resampled dataset's indirect effect and establishes a confidence interval for a specific indirect effect [26]. Data analysis was conducted using SPSS (version 25) and AMOS software. Indices to evaluate the model included chi-square (acceptable when the value is not significant), comparative fit (CFI), nonnormed fit (NNFI; adequate values > .90 and excellent fit > .95), root mean square error of approximation (RMSEA; adequate values < 0.08 and excellent fit < 0.06), and standardized root mean square residual (SRMR < .08) [27]. The level of significance (*p* value) was 5%.

## 3. Results and Discussion

### 3.1. Results

Table 1 presents descriptive statistics and Pearson correlations among the study variables. Significant correlations were found among the study variables *self-efficacy*, *employability skills*, and *role perception* (*r* = 0.16–0.48; *p* < .05–.001).

**Figure 2:**
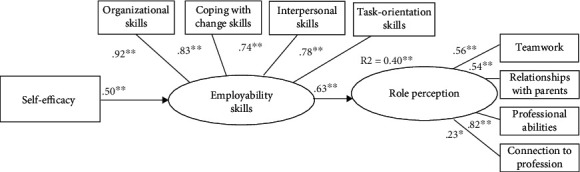
Analysis results of conceptual model mediation. Coefficients in bold are significant at *p* < 0.05.

The SEM provided excellent goodness of fit indices, *χ*^2^(24) = 40.49; *p* = .019; NFI = .93; CFI = .97; RMSEA = .07; SRMR = .05. It explained 40% of the variance in role perception. As depicted in Figure 2, results of this model showed that self-efficacy led to employability skills (*β* = .50, *p* < .001), and the level of employability skills led to role perception (*β* = .62, *p* < .001). An indirect effect was found between self-efficacy and role perception, but the direct effect was not significant (*β* = .024, *p* = .968). This suggests employability skills as a mediator between self-efficacy and role perception.

Figure 2 shows the mediation effect by employability skill.  Table 2 supports Figure 2. It represents the regression coefficients among all model components to describe the size and direction of the relationship between a predictor and the response variable.

### 3.2. Discussion and Implications

This study's main contribution and goal were to examine how self-efficacy and employment-skills variables affect role perception among occupational therapists working in education systems. Applying SEM allows us to isolate both the direct and indirect paths by which self-efficacy affects role perception through employability skills to confirm the proposed conceptual model. Specifically, significant correlations were found among self-efficacy, employability skills, and role perception, with employability skills as the mediator. The model presents appropriate fit indexes, confirming that self-efficacy alone is not enough to develop high and effective role perception.

Based on the significant correlations between the role-perception and employability-skills categories, this study highlights employability skills as a contributor to role perception. For example, teamwork with staff members (expressed as one role-perception factor) correlates with the required abilities to organize and cope with changes. Marks et al. [14] demonstrated that teamwork-process dimensions include adapting to formulate strategy, tracking progress toward task accomplishment, determining deficiencies, providing performance feedback to team members, matching team members' resources to task requirements, regulating the pace of team activities, coordinating those activities' responses, and sequencing and effectively coping with stressful demands and frustration.

High task-orientation skills relate to high-level relationships with parents and vice versa. For instance, family engagement is essential when working with children on individualized bases. Not only are families expert on their children but they also bridge home with school. Families contribute insight and expertise about their child's strengths and challenges. Kennedy et al. [28] explored factors that influence occupational therapists to build relationships with the families of children they service. However, prioritizing relationships with educators, lack of time, and parents' competing demands all influence the occupational therapists' efforts to connect with families and build relationships. These components are at the base of task-orientation skills (i.e., planning and organizing work assignments, accommodating schedules, managing time to complete tasks, and resourcefulness—the ability to function using existing resources).

The correlation of professional abilities and organization skills with role perception's coping-with-change skills is expected. Besides the actual acquisition and retention of a job, Zinser [29] considered communication and interpersonal skills, effective resource management, teamwork, and problem-solving skills as the most important employability elements. Omar et al. [30] indicated that these skills consist of basic, thinking, resource, informational, interpersonal, system and technology, and personal-quality skills.

Our results also confirm the mediating role of employability skills in the relationship between self-efficacy and role perception. Investigating and finding perceived employability as a mediating variable in our model contribute to the literature regarding how self-efficacy relates to employability skills for occupational therapists working in the educational system. Our results confirm and are supported by previous findings that referred to employability skills and self-efficacy as distinct from one another [19].

Self-efficacy is developed in four significant ways—successful experience, social modeling, social persuasion, and individual emotional and physical reactions—with successful experience being the most effective method. Adults reevaluate confidence in their abilities as employment changes occur; strong personal self-efficacy is required to generate the effort to cope with these changes [17].

However, the current study is unique in that it examines a population of occupational therapists within the organization in which they work. Its interesting findings highlight the role of employability skills as a factor to consider in professional-development processes. This factor is crucial to the role perception of occupational therapists in education systems. By thoroughly developing employment skills, occupational therapists can better establish their role and the educational system's expectations for working in teams, working with parents, and working within their abilities.

By raising awareness, these findings can lead to developing employability skills in the clinical-training and professional-development processes over time. These skills are more important in organizations such as education systems, which also require adapting according to the framework's needs. Thus, the results highlight the need to develop various employment skills early, for instance, in the undergraduate training of occupational therapists in general and in the professional development of those in education systems.

### 3.3. Limitations and Future Studies

Because this study used a convenience sample, many participants were engaged in consulting roles in the education system; thus, their self-efficacy, employment skills, and role perception may be higher than average. Further research examining a larger sample could provide additional insight. Occupational therapists in education systems work simultaneously in different settings, and each setting may require additional skills that may have contributed to the participants' perceptions of their roles. For instance, the children's ages and population types may contribute to or delay the occupational therapists' role perception. Further research could separate occupational therapists who work in clinics from those who work in kindergartens or schools or split the types of populations with which the occupational therapists work.

Despite these limitations and the need for further research, it can be argued that in all the ages and population types with which occupational therapists in education systems work, these occupational therapists need to develop employment skills to meet the organization's demands in general and the profession's demands in particular. Professional training that includes the practice of these employability skills may be an effective and proactive scaffold. Finally, future studies should consider the results for international relevance.

### 3.4. Implications for Occupational Therapy Practice

Occupational therapy practitioners in school systems have the unique ability to see children in a natural context within the school, where children make friends and participate in many activities. These occupational therapists can improve their own abilities by recognizing role-perception components and each year choosing to enhance some elements that challenge them. They can raise their self-awareness of the educational system's employability requirements by performing indepth activity analysis and improving employability skills through professional advancement courses across disciplines (e.g., lectures from marketing, management, and communications).

## 4. Conclusions

This study addresses four significant components of the role perception of occupational therapists working in education systems. Self-efficacy, a mediating component, and employment skills influence role perception of occupational therapists working in education systems; thus, employment skills should be included in professional training and development courses. The study's contributions to the professional literature are its consideration of general employability skills as a significant component of the role perception of occupational therapists in the educational system and the ability to use this information to create an effective program for professional development.

## Figures and Tables

**Table 1:** Means, standard deviations, and correlations among study variables.

	Variable	*M*	SD	1	2	3	4	5	6	7	8
1.	Self-efficacy	3.96	.44								
2.	Employability skills: organizational skills	5.59	.74	.47^∗∗∗^							
3.	Employability skills: interpersonal skills	6.34	.51	.35^∗∗∗^	.66^∗∗∗^						
4.	Employability skills: coping with change skills	6.10	.62	.36^∗∗∗^	.76^∗∗∗^	.77^∗∗∗^					
5.	Employability skills: task-orientation skills	6.01	.76	.41^∗∗∗^	.71^∗∗∗^	.65^∗∗∗^	.65^∗∗∗^				
6.	Role perception: teamwork	3.26	.44	.31^∗∗∗^	.36^∗∗∗^	.27^∗∗^	.38^∗∗∗^	.29^∗∗∗^			
7.	Role perception: relationships with parents	4.57	1.22	.15	.27^∗∗∗^	.19^∗^	.16^∗^	.31^∗∗∗^	.26^∗∗^		
8.	Role perception: professional abilities	5.77	.97	.23^∗∗^	.48^∗∗∗^	.35^∗∗∗^	.43^∗∗∗^	.32^∗∗∗^	.46^∗∗∗^	.47^∗∗∗^	
9.	Role perception: connection to the profession	5.19	1.21	.15	.18^∗^	.27^∗∗^	.25^∗∗^	.17^∗^	.09	.17^∗^	.16

^∗^ *p* < .05, ^∗∗^*p* < .01, ^∗∗∗^*p* < .001.

**Table 2:** Model coefficients.

Variable		Coefficient	Estimate	*p*
Employability skills	<---	Self-efficacy	.498	^∗∗∗^
Role perception	<---	Employability skills	.617	^∗∗∗^
Role perception	<---	Self-efficacy	.024	.806
Role perception: teamwork	<---	Role perception	.561	
Role perception: relationships with parents	<---	Role perception	.542	^∗∗∗^
Role perception: professional abilities	<---	Role perception	.818	^∗∗∗^
Role perception: connection to the profession	<---	Role perception	.231	.018
Employability skills: organizational skills	<---	Employability skills	.917	
Employability skills: interpersonal skills	<---	Employability skills	.740	^∗∗∗^
Employability skills: copping with change	<---	Employability skills	.827	^∗∗∗^
Employability skills: task-orientation skills	<---	Employability skills	.782	^∗∗∗^

^∗∗^ *p* < .01; ^∗∗∗^*p* < .001.

## Data Availability

The data used to support the findings of this study are restricted by the Ethics Committee (AU-HEA-YF-20191030) and the Education Ministry Ethics Committee (No. 1000 990) that approved this study in order to protect participant's privacy. Data are available from Dr. Yael Fogel for researchers who meet the criteria for access to confidential data.
